# School Anxiety Accommodation in Youth: Prevalence and Patterns Among Teachers

**DOI:** 10.1007/s10578-025-01853-z

**Published:** 2025-05-24

**Authors:** Åshild Tellefsen Håland, Thomas B. Bertelsen

**Affiliations:** Sørlandet Sykehus Kristiandsand, University of Agder, Kristiansand, 4630 Norway

**Keywords:** Anxiety accommodation, Teacher accommodation, Classroom management, Youth anxiety

## Abstract

This study addressed the pressing need to investigate the prevalence and nature of teachers’ anxiety-accommodating behaviors across different grades. Employing a cross-sectional design, we disseminated a questionnaire to 1200 (243 responded) teachers within the Kristiansand municipality in Norway, spanning elementary to high schools. The findings revealed a pronounced trend: every teacher reported at least one anxiety-accommodating behavior, with 71% indicating frequent use. Factor analysis further elucidated two primary accommodation strategies: ‘Modification’ and ‘Participation’, which had distinct correlations to the grade levels taught. These findings fit with parental accommodation patterns for anxiety observed in the community, suggesting similar accommodation behaviors among community members and teachers. Given this widespread prevalence of anxiety accommodation, coupled with the limited availability of school-based CBT interventions, this study revealed a significant divergence from evidence-based practices that focus primarily on confronting anxiety provoking situations. The potential biases of self-reporting and the study’s regional focus stand as limitations. However, the clear implication is the urgent need for tailored interventions aimed at reducing anxiety accommodating behaviors for educators, paralleling similar strategies designed for parents with the goal of improving the way school personnel manage and help to mitigate student anxiety in the school environment.

## Introduction

Cognitive behavioral therapy (CBT), including school-based CBT, has been shown to be efficacious for the treatment of anxiety disorders in children and adolescents [18]. A central component in CBT for anxiety is to expose yourself to what you fear without avoiding situations or using safety behaviors [19]. While exposure is a crucial component in CBT, enabling individuals to face their fears head-on, many schools do not use an exposure-informed approach to combat avoidance in their anxious students. Despite the effectiveness of CBT, most schools neither employ school-based CBT interventions for anxious students nor do they establish general guidelines on how school staff should support anxious and avoidant students [18]. In fact, contrary to evidence-based approaches focused on confronting anxiety evoking situations, many teachers respond to anxious students with both subtle and overt suggestions to avoid challenging situations [5, 6]. Typical examples of school staff-led accommodation include exempting socially anxious students from speaking up in class, giving presentations or setting policies that allow students to leave the classroom whenever they wish.

Accommodation in a school setting may be termed *school-based accommodation* which can be understood to be similar to the concept of *parental accommodation.* School-based accommodation, like parental accommodation, refers to modifying behaviors to temporarily alleviate a child’s anxiety (e.g., exempting students from speaking in class). Since accommodations relieve anxiety they may be an important temporary tool to ensure students can participate in school and accommodations may sometimes be legally mandated (e.g., through IEPs or 504 plans in the US) [11]. However, accommodations should be considered temporary tools because while they may provide immediate relief, accommodations can inadvertently intensify or prolong the anxiety over time [12]. Accommodation conflicts with the emphasis CBT places on confronting avoidance and facing feared stimuli, and thus could impede a youth’s ability to benefit from CBT treatment.

Though there’s abundant research on parental accommodation (For overview see [8], studies on school-based accommodations for anxious students are limited. Knowledge of the extent and nature of school accommodation for anxiety is insufficient. Only four studies have examined the nature of school-based accommodation related to anxious children [5–7, 16].

Within the existing research, Green et al. [7] delved into the realm of school functioning and the provision of school-based supports for anxious youths, examining a sample of 51 youths and their mothers at an outpatient clinic. Utilizing the School Resources Survey-Anxiety (SRS-A), specifically designed for this study, they assessed anxiety-related experiences and accommodations, like extended test times and permission for classroom exits. They found that a majority of the participants received at least one form of school accommodation, which promoted avoidance of anxiety-inducing situations. Complementing this, Conroy et al. [5] surveyed 315 school staff members to understand their approach to student anxiety accommodations. Their results revealed a dichotomy: while some accommodations were approach-oriented, a significant portion (92.5%) of teachers used at least one accommodation that promoted avoidance of anxiety, such as allowing students to not participate in class activities.

Building on these findings, Phillips et al. [16] investigated accommodation behaviors in schools for 74 youths diagnosed with anxiety disorders. Through analyzing school documents rated by youth anxiety experts, they found an average of over 20 accommodation behaviors per child, with notable variation based on the type of anxiety disorder. Youths with social anxiety disorder were more likely to receive accommodations that promoted avoidance whereas those with specific phobias tended to receive more approach-oriented supports. Similarly, Ginsburg et al. [6] focused on the frequency of accommodation behaviors among teachers, noting that academic performances were commonly associated with accommodation. Their study highlighted a significant correlation between teachers’ accommodation behaviors and increased student avoidance and anxiety severity, suggesting a complex relationship between educational practices and the management of student anxiety. These studies collectively point to a nuanced landscape of school-based accommodations for anxiety, revealing a mix of approach and avoidance strategies with varying degrees of alignment with students’ needs.

The existing literature on school accommodations for student anxiety, while revealing, is marked by several limitations that highlight the need for more comprehensive research. Studies like those of Phillips et al. [16] and Green et al. [7] were limited by small sample sizes and a reliance on formal documents and self-reports. These studies were also limited in their generalizability due to gathering data from individuals in an outpatient clinical setting. Conroy et al. [5] faced potential biases by focusing using a sample consisting of teachers who self-reported that they identified pupil anxiety as a primary concern in their classroom. Ginsburg et al. [6] provided insights into the high prevalence of inappropriate accommodations for anxious students among primary school teachers and noted a correlation between increased teacher accommodations and heightened student anxiety, similar to parental accommodation patterns. However, their study, along with others, was limited in scope, focusing only on children from elementary schools, using a sample solely made up of anxious students, or teachers recognizing anxiety as a significant classroom issue. Notably, there appears to be a lack of research addressing how general teacher populations in various educational settings interact with and accommodate students who avoid anxiety-provoking situations, highlighting a gap in understanding the broader context of anxiety management in educational environments.

The prevailing research on accommodations for student anxiety within schools is still being developed and there are several limitations that our study seeks to address. One limitation is that the existing studies used different measures since no questionnaire has yet been designed specifically for assessing anxiety accommodation in school. Furthermore, the narrow focus of previous studies on specific populations (e.g., including only students identified by staff as anxious) and the lack of questionnaires specifically designed to address school accommodation have left a gap in our understanding of how the general teacher population manages student anxiety throughout a typical school day. This gap is particularly pronounced when considering the potential for school-based accommodations to exacerbate anxiety symptoms over time. By extending our analysis to a more diverse and representative sample of teachers, and laying the foundation for a questionnaire aimed specifically at school-based accommodation, our research aims to uncover the prevalence and typology of school-based accommodations, providing a clearer picture of current practices and the motivations behind them.

The ultimate goal is to inform the development of effective strategies that can mitigate the use of potentially maladaptive anxiety accommodations. Recognizing that children spend a substantial portion of their day within the educational environment, the implications of our findings are far-reaching. By identifying the patterns and structures of accommodations, we can begin to pave the way for interventions that encourage teachers and school personnel to support students in confronting, rather than avoiding, anxiety-provoking situations. This shift is critical, as it aligns with the principles of CBT and has the potential to reduce the long-term incidence of anxiety problems among students. Moreover, enhancing teacher and staff competence in CBT principles could have a synergistic effect, improving outcomes for students who are concurrently receiving outpatient treatment for anxiety. Our study, therefore, not only aims to fill the current research void but also to contribute to a paradigm shift in how educational settings can play an active role in the treatment and management of student anxiety.

In keeping with this goal, we present the following research questions:

1. What is the prevalence of anxiety-accommodating behaviors among teachers?
2. What are the most common accommodating behaviors delivered by teachers?
3. What is the factor structure of the measure of teachers’ reported accommodation strategies used in this study?

## Method

### Design and Participants

This study utilized a cross-sectional design, employing a questionnaire distributed to teachers within the municipality of Kristiansand. The questionnaire was digitally disseminated through a Facebook group specifically targeted towards these educators. Teachers participating in this study were employed at schools within the municipality of Kristiansand. The questionnaire reached educators from 248 schools, and we received responses from teachers across 61 of these institutions. In total, 243 of 1200 teachers in the Facebook group participated in the survey. Their distribution across the various school levels was as follows: Elementary schools: *n* = 75 (30.74%), Middle schools: *n* = 73 (29.92%), High schools: *n* = 95 (38.93%). On average, each school had approximately 4.63 teachers participating in the study (*SD* = 2.98), with a range from 1 to 13 teachers per school. These schools catered to student populations varying from 200 to 641 pupils. Collectively, the schools within Kristiansand municipality serve an overall student body of 20,143, primarily composed of ethnic Norwegians in an urban environment.

### Measures

The questionnaire used for this study used questions from several sources and adapted them to be relevant to the school setting. Questions were adapted from Family Accommodation questionnaire [14], Parental Accommodation Scale [2] and clinical experience as well as feedback from teachers. The delivered questionnaire included 26 questions related to frequency of different types of school accommodation. These questions asked, “How often does the following accommodations to anxious students occur in your classroom”, and were responded to on a five-point scale (1 = Never, 2 = Rarely, 3 = Sometimes, 4 = Often, 5 = Very often). Additionally, we included 8 questions to explore potential reasons given for accommodating behaviors by teachers, which were included as an exploratory aspect.

### Data Analysis

All analyses were conducted in JASP and using the R statistical software [10, 17]. For all analyses assumptions of normality and absence of outliers were investigated using Shapiro-Wilks test and Cooks Distance, indicating that assumptions were met for all analyses. There was a low degree of missing information (0.41%, 1 question not filled), and all analyses were conducted with complete cases in line with recommendations for analyzing when small amounts of data are missing [9].

The data analysis strategy was to first analyze which accommodation behaviors were most frequently reported as present by teachers and which accommodation behaviors were reported as most frequently used when teachers utilized them. The next step of the data analysis was conducting factor analysis on the reported school accommodation behaviors. This involved assessment of several assumptions: (1) The sample size was deemed adequate given that we had 26 questions and 243 respondents which is in line with the recommended 5–10 participants per item [15]. (2) The observations were deemed independent based on investigation on clustering per school which did not show a specific pattern. However, it should still be noted that almost all schools gave responses from several teachers. (3) Based on exploratory data analysis, variables were deemed to be linearly related and multivariate normal and there were no signs of perfect multicollinearity between observed factor loadings. The observed responses to accommodating behavior were entered in an exploratory factor analysis using promax rotation. Based on this we removed observed variables that had loading lower than 0.40 or had cross-loadings higher than 0.2, as recommended [1]. The final step of the analysis was to analyze correlations between different types of accommodation and whether these were related to suspected reasons for accommodating or interventions suspected to reduce accommodation.

## Results

All respondents had at least one accommodating behavior, and 71% of teachers reported using at least one accommodating behavior “Often” or “Very Often”. The average number of behaviors reported as “Often” or “Very often” per teacher was 3.60 (*SD* = 3.98, min = 0, max = 22). The behaviors most teachers reported doing Sometimes, Often or Very often were the following: “Students can leave the classroom when they feel anxious” (68%), “Students have special arrangements regarding their seating in the class” (66%), “Have you helped the student(s) outside of your regular working hours (stayed longer, arrived early, answered emails, taken calls) to accommodate their anxiety” (63%), “Students can go to a room to be alone” (62%), “Students are exempted from oral presentations in front of the class” (60%). The behavior that was most frequently reported as “Very often” used was the following: “Students are excused from showering at school?” (15.19%), “Students can leave the classroom when they feel anxious” (10.2%), “Special routines/rules are established for anxious students” (10%), “How often do you provide reassurance or comfort to anxious students” (9%), “Students have special arrangements regarding their seating in the class” (9%), “Have you helped the student(s) outside of your regular working hours (stayed longer, arrived early, answered emails, taken calls) to accommodate their anxiety” (8%). Overview of frequency of accommodating behavior can be found in Table 1.

**Table 1:** Frequency of school accommodation for anxiety

Questionnaire items	Mean (SD)	Q_1_	Q_3_
1. Students can leave the classroom when they feel anxious	2.98 (1.22)	2	4
2. Teacher accompanies students to the restroom	1.67 (0.84)	1	2
3. Teacher accompanies students for parts or the entire journey from home to school	1.45 (0.79)	1	2
4. Students are exempted from physical education	2.05 (0.96)	1	3
5. Students are exempted from oral presentations in front of the class	2.76 (1.09)	2	3
6. Students are exempted from taking tests and exams with other students in the class	2.23 (1.02)	1	3
7. Students sit in a separate room during break time	1.97 (0.89)	1	3
8. Students are exempted from working with other students	1.86 (0.88)	1	2
9. Students receive instruction outside the classroom due to anxiety	1.91 (0.97)	1	3
10. Students have an agreement not to be asked anything in class	2.26 (1.04)	1	3
11. Students’ parents are allowed to be present in the classroom	1.33 (0.70)	1	1
12. Students’ parents have an agreement to wait outside the classroom for a while before leaving the school	1.36 (0.67)	1	2
13. Teacher picks up individual students in the schoolyard in the morning before school starts	2.05 (1.17)	1	3
14. Students are excused from showering at school	2.72 (1.35)	2	4
15. Students eat in a separate room	1.75 (0.89)	1	2
16. Students can go to a room to be alone	2.71 (1.04)	2	3
17. Students have special arrangements regarding their seating in the class	2.94 (1.17)	2	4
18. Students can go home when it suits them	1.71 (0.86)	1	2
19. Special routines/rules are established for anxious students	3.13 (1.07)	2	4
20. How often do you provide reassurance or comfort to anxious students	3.23 (0.98)	3	4
21. Have you avoided doing things in the class because of some students’ anxiety	2.42 (1.03)	2	3
22. Have you changed class routines due to students’ anxiety	2.38 (0.97)	2	3
23. Have you done things that would normally be the students’ responsibility	2.48 (0.98)	2	3
24. Have you helped the student(s) outside of your regular working hours (stayed longer, arrived early, answered emails, taken calls) to accommodate their anxiety	2.86 (1.13)	2	4
25. Have you changed your leisure plans to accommodate anxious students	1.61 (0.78)	1	2
26. How often do you assist students in avoiding things that could potentially make them more anxious	2.43 (0.96)	2	3

Q_1_ = 25 th percentile, Q_3_ = 75 th percentile. These questions asked “How often does the following accommodations to anxious students occur in your classroom”, and were responded to on a five-point scale (1 = Never, 2 = Rarely, 3 = Sometimes, 4 = Often, 5 = Very often)

**Table 2:** Correlation between accommodating behaviors

	Q.1	Q.2	Q.3	Q.4	Q.5	Q.6	Q.7	Q.8	Q.9	Q.10	Q.11	Q.12	Q.13
Q.1	-												
Q.2	0.26	-											
Q.3	0.28	0.30	-										
Q.4	0.56	0.16	0.35	-									
Q.5	0.50	0.13	0.26	0.57	-								
Q.6	0.63	0.14	0.25	0.54	0.59	-							
Q.7	0.53	0.17	0.34	0.50	0.42	0.54	-						
Q.8	0.43	0.22	0.18	0.50	0.38	0.54	0.57	-					
Q.9	0.45	0.09†	0.30	0.51	0.46	0.55	0.49	0.58	-				
Q.10	0.48	0.07†	0.25	0.49	0.62	0.54	0.44	0.39	0.48	-			
Q.11	0.23	0.35	0.21	0.24	0.23	0.19	0.14	0.21	0.26	0.16	-		
Q.12	0.20	0.39	0.26	0.22	0.16	0.12	0.17	0.19	0.17	0.18	0.53	-	
Q.13	0.35	0.28	0.53	0.31	0.27	0.30	0.28	0.29	0.28	0.25	0.30	0.41	-
Q.14	0.42	0.04†	0.30	0.52	0.53	0.46	0.40	0.36	0.44	0.50	0.20	0.14	0.37
Q.15	0.45	0.19	0.26	0.39	0.34	0.44	0.52	0.52	0.50	0.39	0.22	0.25	0.36
Q.16	0.57	0.28	0.31	0.51	0.43	0.55	0.52	0.54	0.54	0.46	0.24	0.18	0.46
Q.17	0.48	0.24	0.23	0.51	0.54	0.55	0.46	0.47	0.49	0.60	0.15	0.15	0.34
Q.18	0.49	−0.04†	0.23	0.54	0.54	0.49	0.52	0.42	0.45	0.55	0.26	0.27	0.29
Q.19	0.54	0.22	0.39	0.53	0.45	0.54	0.43	0.45	0.51	0.46	0.19	0.20	0.45
Q.20	0.46	0.30	0.34	0.34	0.30	0.41	0.33	0.28	0.44	0.28	0.22	0.24	0.50
Q.21	0.45	0.11†	0.30	0.44	0.39	0.41	0.40	0.41	0.50	0.47	0.21	0.15	0.35
Q.22	0.47	0.19	0.27	0.51	0.40	0.49	0.47	0.49	0.57	0.43	0.20	0.20	0.42
Q.23	0.41	0.29	0.17	0.34	0.31	0.37	0.37	0.45	0.46	0.36	0.22	0.13	0.35
Q.24	0.49	0.22	0.30	0.39	0.35	0.48	0.39	0.36	0.49	0.40	0.19	0.20	0.49
Q.25	0.33	0.20	0.21	0.35	0.21	0.33	0.24	0.31	0.36	0.31	0.16	0.18	0.35
Q.26	0.39	0.18	0.21	0.46	0.42	0.47	0.42	0.42	0.48	0.41	0.08	0.16	0.32

	**Q.14**	**Q.15**	**Q.16**	**Q.17**	**Q.18**	**Q.19**	**Q.20**	**Q.21**	**Q.22**	**Q.23**	**Q.24**	**Q.25**	**Q.26**
Q.14	-												
Q.15	0.45	-											
Q.16	0.47	0.46	-										
Q.17	0.44	0.44	0.61	-									
Q.18	0.43	0.52	0.40	0.46	-								
Q.19	0.47	0.45	0.59	0.58	0.47	-							
Q.20	0.44	0.38	0.47	0.41	0.27	0.57	-						
Q.21	0.36	0.39	0.48	0.51	0.38	0.50	0.50	-					
Q.22	0.32	0.38	0.56	0.57	0.39	0.57	0.54	0.72	-				
Q.23	0.35	0.42	0.53	0.52	0.34	0.46	0.46	0.49	0.58	-			
Q.24	0.42	0.41	0.50	0.54	0.33	0.54	0.65	0.53	0.62	0.53	-		
Q.25	0.35	0.30	0.37	0.33	0.23	0.36	0.40	0.36	0.39	0.33	0.59	-	
Q.26	0.34	0.35	0.50	0.52	0.40	0.55	0.45	0.45	0.56	0.49	0.55	0.36	-

Q. denotes the questionnaisre item which can be found in Table 1. All Correlations are significant except those denoted by †

### Factor Analysis

With the exception of questionnaire item 2 (“Teacher accompanies students to the restroom”) all questions were significantly correlated as seen in Table 2. Specifically, questionnaire item 2 was not significantly correlated with the following other items: “Students receive instruction outside the classroom due to anxiety?”, “Students have an agreement not to be asked anything in class?”, “Students are excused from showering at school?”, “Students can go home when it suits them?” and “Have you avoided doing things in the class because of some students’ anxiety?”.

The first step in the factor analysis resulted in a two-factor solution with a RMSEA of 0.08 (90% CI [0.07, 0.09]), CFI = 0.85 and BIC = 763.09. This model included all 26 variables and was a two-factor solution. However, 14 variables had low loadings (< 0.40) and or high cross loadings (> 0.20). After removing these, the second model included 12 variables and resulted in an improved fit on the RMSEA of 0.06 (90% CI [0.04, 0.08], CFI = 0.96 and BIC = −149. The second model maintained the two-factor solution and was considered the final model. The two factors in the final model were: Factor 1 (Modification) and Factor 2 (Participation). The two factors were correlated (*r* = 0.45) and had Eigenvalues of 4.98 and 1.71 respectively. As a supplemental analysis, the two factors were investigated for their correlation with which grade the teacher taught (Elementary, Middle school or High school). Factor 1 was positively correlated with higher grades (*r* = 0.54, *p* < 0.001), whereas Factor 2 was negatively correlated with higher grades (*r* = −0.13, *p* = 0.04). An overview of the final model can be found in Table 3.

**Table 3:** Overview of factor model

Questionnaire item	Factor 1	Factor 2	Uniqueness
6. Students are exempted from taking tests and exams with other students in the class	0.81		0.40
4. Students are exempted from physical education	0.73		0.45
9. Students receive instruction outside the classroom due to anxiety	0.72		0.49
5. Students are exempted from oral presentations in front of the class	0.71		0.50
17. Students have special arrangements regarding their seating in the class	0.70		0.51
7. Students sit in a separate room during break time	0.69		0.54
8. Students are exempted from working with other students	0.66		0.53
14. Students are excused from showering at school	0.65		0.59
13. Teacher picks up individual students in the schoolyard in the morning before school starts		0.41	0.68
12. Students’ parents have an agreement to wait outside the classroom for a while before leaving the school		0.87	0.33
11. Students’ parents are allowed to be present in the classroom		0.64	0.58
2. Teacher accompanies students to the restroom		0.52	0.73

Factor 1 is interpreted as related to modification of school activities, whereas Factor 2 is interpreted as Participation since it requires active participation by teachers or parents

### Exploratory Analysis

As an exploratory part of our study, we investigated potential reasons why teachers might employ certain unhelpful accommodation. Of particular note, the majority of teachers reported that the following questionnaire items were not true or only slightly true: 27. I give in to pressure from parents to accommodate for anxiety (73.02%), 28 I struggle with anxiety, which makes it difficult for me to help pupils approach their anxiety (97.13%). On the other hand, teachers responded that they found the following questionnaire items moderately true or very true: 29. There is not enough time and/or resources to work systematically with avoiding accommodation for anxious pupils (65.00%), 31. The school’s management has a good understanding of how to help anxious pupils (84.58%). The remaining questions did not have a clear majority for teachers responding true or not true, but are presented in Table 4.

**Table 4:** Questionnaire items related to potential reasons for accommodation

Questionnaire item	Not true	Slightly true	Moderately true	Very true
27. I give in to pressure from parents to accommodate for anxiety.	37.34%	35.68%	24.48%	2.49%
28.I struggle with anxiety, which makes it difficult for me to help pupils approach their anxiety.	89.34%	7.79%	1.66%	0%
29. There is not enough time and/or resources to work systematically with avoiding accommodation for anxious pupils.	19.17%	15.83%	41.67%	23.33%
30. I have high competence in helping with anxious pupils	14.46%	28.10%	44.63%	12.81%
31. The schools management has a good understanding of how to help anxious pupils	5.00%	10.42%	62.50%	22.08%
32. I am afraid of causing increased anxiety or abseentism if i dont accommodate for anxiety	23.43%	26.36%	36.40%	13.81%
33 I am confused by contradictory advice about how to help anxious pupils	30.38%	26.16%	33.76%	9.70%
34. In my class we have systematic and goal-oriented plans for how pupils can gain mastery over their anxiety	18.91%	18.91%	34.87%	27.31%^a^

Question 34 was assessed using a five-category rating, in this presentation the final two categories are presented together for enhanced readability

## Discussion

The present study sought to discern the prevalence and nature of school accommodation for anxiety. The findings, derived from a sample of 243 teachers spanning elementary to high schools, are striking. Our investigation indicated that every teacher reported at least one anxiety accommodating behavior, with a notable 71% indicating regular use of accommodation. On average, teachers implemented 3.60 accommodation behaviors frequently, suggesting a multifaceted pattern of school accommodation. This is further underscored by the emergence of a two-factor model from the responses, highlighting strategies categorized as making modifications to avoid anxiety (e.g. being exempt from certain activities) and having adults participate to avoid anxiety (e.g. allowing parents to be present in the classroom, teacher accompanying student to bathroom).

In light of our main finding that every teacher reported at least one anxiety-accommodating behavior, it’s evident that many schools currently lack a structured approach to addressing avoidance among their anxious students, despite the established efficacy of exposure-based CBT for childhood and adolescent anxiety disorders. This aligns with concerns raised by [18], pointing to a significant gap in school-based CBT interventions and general guidelines for assisting these students. These findings are unfortunately in line with the described prevalent culture of teacher-led accommodations, as highlighted by [5, 6]. These findings are also in line with Green et al. [7, 16] and indicate a troubling divergence from evidence-based practices. While this trend might stem from potential knowledge gaps regarding CBT principles, as suggested by Ginsburg et al. [6], our exploratory findings suggest time and resource constraints may be more influential than lack of knowledge. Nonetheless, the findings from this study highlight a pressing need for interventions to reduce unhelpful school-accommodations for anxiety.

Another important finding from the study is the identification of two predominant teacher-led accommodation strategies: ‘Modification’ and ‘Participation’, with distinct correlations to the grade levels taught. This factor model is similar to the one found in the FASA [14] and PAS [2], which were developed to investigate parental accommodation for anxiety. These findings suggest that teachers may act similarly to parents when they accommodate for anxiety - attempting to help youth with short term relief from anxiety, despite the risk of increasing it over time [12]. This is in line with our own experiences from a previous project (Bertelsen, Wergeland, et al., [4]), where we have seen that teachers act similarly to parents with respect to when and how they accommodate anxious youth. Encouragingly, given the existence of potent interventions addressing parental accommodation in youth anxiety [3, 13], our findings suggest the potential of crafting analogous strategies tailored for educators, potentially offering a robust remedy for anxious students.

Our exploratory research suggests that the reasons behind teachers’ accommodation behaviors for student anxiety are less influenced by external pressures from parents or personal challenges, differing from parental accommodation patterns [8]. Teacher accommodation appears to be particularly influenced by systemic issues within schools, such as resource constraints and time limitations. This finding fits with concerns in existing literature about the misalignment of school practices with evidence-based approaches like CBT [18]. A significant portion of teachers (65.00%) indicated a lack of time and resources as a reason for accommodation, resonating with studies by Ginsburg et al. [5, 6], which highlighted the absence of school-based CBT interventions and guidelines for supporting anxious students. Furthermore, the majority of teachers believe their school management understands how to help anxious pupils, yet this belief contrasts with the prevalent use of avoidance-based accommodations, indicating a gap between effective anxiety management strategies and current school practices. This discrepancy has also been noted in the studies by Green et al. [7, 16]. An important consideration in understanding this discrepancy is that accommodation is complex in the sense that it may be unhelpful in the long term but it may be beneficial in the short term and sometimes even legally mandated (e.g., via IEPs or 504; Kagan et al., [11]. When faced with time and resource constraints, the task of implementing nuanced accommodation strategies may become difficult and the focus may shift toward dealing with immediate distress. This underscores the need for nuanced evidence-based approaches to accommodation in the educational settings.

Although the findings in this study reveal important areas for attention in school settings related to student anxiety, certain limitations are noted. First, the study’s scope was limited to a specific part of Norway. Given the pivotal role that the school day plays in socialization within the Norwegian context, it’s uncertain if the findings would resonate in diverse cultural or educational settings. Another limitation concerns the reliance on teacher self-reports. The potential biases inherent in such data collection means teachers might overreport accommodating behaviors when stressed or underreport in an attempt to project competence. Likewise our sampling method does not allow us to understand differences between those that completed the questionnaire and those that did not. Lastly, our study does not explore the possible link between teacher accommodation and student anxiety levels. Prior research indicates that heightened anxiety in youth could amplify adult accommodating behaviors [3], which could confound our findings. Such a relationship, if present, could offer more nuanced insights into the dynamics of teacher-student interactions in the context of anxiety.

In conclusion, the pervasiveness of anxiety-accommodating behaviors among teachers is alarmingly apparent, with every educator in our sample reporting at least one such practice. These behaviors, encompassing both passive and active avoidance strategies, underline a concerning shift away from evidence-based approaches that emphasize facing fear as opposed to avoiding it. The findings suggest that teachers, much like parents, may prioritize immediate relief for anxious students, even if it inadvertently exacerbates the anxiety in the long run. Despite the study’s regional focus and potential biases from self-reporting, the implications are profound: there exists an urgent need for interventions designed to curb unhelpful school accommodations for anxiety. By bridging the knowledge gap and introducing strategies akin to those used with parents, educators could be better equipped to manage and alleviate student anxiety, ultimately fostering a more supportive and effective learning environment.

## Data Availability

No datasets were generated or analysed during the current study.
