# Correction to: School Anxiety Accommodation in Youth: Prevalence and Patterns Among Teachers

**DOI:** 10.1007/s10578-025-01874-8

**Published:** 2025-06-19

**Authors:** Åshild Tellefsen Håland, Thomas B. Bertelsen

**Affiliations:** Sørlandet Sykehus Kristiandsand, University of Agder, Kristiansand, 4630 Norway

**Correction to: Child Psychiatry & Human Development** 10.1007/s10578-025-01853-z

In this article, under section “Methods” and under heading “Design and Participants”, the sentence beginning with ‘The questionnaire reached educators…’, the value 248 should have read as 48.

The complete sentences should have read as follows: “The questionnaire was digitally disseminated through a link sent to these educators. Teachers participating in this study were employed at schools within the municipality of Kristiansand. The questionnaire reached educators from 48 schools. In total, 243 of 1200 teachers in the Facebook group participated in the survey.

